# Typical clinical manifestations of anxiety and depression in patients with medical illness and general notions about their treatment

**DOI:** 10.1192/j.eurpsy.2025.174

**Published:** 2025-08-26

**Authors:** L. Bartova

**Affiliations:** Department of Psychiatry and Psychotherapy, Clinical Devision of General Psychiatry, Medical University of Vienna, Vienna, Austria

## Abstract

**Abstract:**

Anxiety and depression are common psychiatric comorbidities in chronic medical illnesses, such as cardiovascular, endocrine, and neurological diseases. These conditions are associated with greater disease severity, poorer functional outcomes, and reduced quality of life for patients and their families. Despite their significant global burden, they are often underdiagnosed due to overlapping symptoms with primary medical conditions. Symptoms in medically ill patients may differ from classic psychiatric presentations, often involving heterogeneous (psycho)somatic phenomena like fatigue, autonomic irregularities, gastrointestinal symptoms, sleep disturbances, and cognitive impairments. Anxiety typically manifests as hypervigilance, excessive worry, and irritability, while depression presents with persistent sadness, psychomotor slowing, and loss of motivation, pleasure, and interest. Subsyndromal symptoms, though often untreated, can exacerbate functional decline and disease progression. Effective management requires a multimodal approach, with modern psychopharmacotherapy as a cornerstone. Selective Serotonin Reuptake Inhibitors (SSRIs) and Serotonin-Norepinephrine Reuptake Inhibitors (SNRIs) are recommended first-line treatments due to their safety and efficacy. In case of insufficient response, add-on strategies may include second-generation antipsychotics, lithium, esketamine, or pregabalin for predominant anxiety. Emerging evidence supports the use of phytotherapeutics as Silexan and Rhodiola rosea for subsyndromal symptoms. Non-pharmacological interventions also play an important role. Cognitive Behavioral Therapy remains the first-line psychotherapeutic option, complemented by mindfulness techniques, psychoeducation, biofeedback, and relaxation strategies, particularly for anxiety. Chronobiological treatments like bright-light therapy and sleep deprivation have proven effective for depression, especially in seasonal patterns. Brain stimulation methods, including transcranial magnetic stimulation and electroconvulsive therapy, may also be considered based on individual needs. Importantly, successful physician-patient interactions, directly and promptly addressing emotional and psychological concerns during consultations and daily rounds, are crucial in fostering treatment adherence and improving outcomes. In conclusion, anxiety and depression in medically ill patients present unique diagnostic and therapeutic challenges. Early recognition and individualized, multimodal treatment strategies can enhance adherence and outcomes. Integrating pharmacological and non-pharmacological approaches within collaborative care models and recommended treatment algorithms is essential for effectively addressing individual clinical presentations.

**Disclosure of Interest:**

None Declared

